# The History, Diagnosis, and Treatment of the Fevers of the United States

**Published:** 1848-06

**Authors:** 


ART. IV.—*The History, Diagnosis, and Treatment of the Fevers of the United States.* By ELISHA BARTLETT, M. D., Prof. of the Theory and Practice of Physic in the Medical department of Transylvania University. Philadelphia, Lea & Blanchard, 1847. 8vo. pp. 547.

This work is, in part, a second edition of a publication issued some five or six years ago, which was entitled an essay on Typhoid and Typhus Fevers, being restricted in its scope to the fevers just named. The present publication embraces, not only new matter relating to these fevers, but an account of the periodical fevers of the United States, viz: Intermittent and Remittent fevers, and, also, Yellow Fever. These additions, as the author remarks, entitle the present volume to be considered a new work, rather than a new edition of the first publication, the size of the former being double that of the latter, and he submits it, in its present shape, as a "systematic and methodical treatise on the fevers of the United States."

It is refreshing in these days of translations, and republications of Foreign productions, to meet with a *bona fide American* book. We may add that it is also a source of comfort to feel assured that an author has not appropriated extracts from an unpublished French treatise, giving an almost literal translation, without credit, or reference to the work upon which the depredation was committed. We do not mean to insinuate that such instances of literary larceny are common, but the remark is not aimless: let those flutter who are conscious of being hit. If we are not deceived by an intensity of desire to see our expectations fulfilled, the fruits of the new spirit which has of late been infused into the medical profession of this country, will be apparent in creditable additions to our national medical literature. Already announcements of several works in preparation have been made from different quarters. Successful examples will stimulate and encourage others, and we venture to predict that it will not be long ere American publishers will find it to be both less acceptable, and less profitable to reproduce foreign works, enlarged and improved by the labors of American editors, than to furnish publications exclusively American. The time will come, we trust, when the sneering question "who reads an American book?" will not be appropriate on our own soil, albeit, this contemptuous remark by a foreign reviewer may express the prevailing sentiment of his own countrymen.

After giving utterance to the foregoing sentiments, it is needless to say that we greet with pleasure the work, the title page of which we have placed at the head of this article.

The previous productions of Prof. B. have secured for him a high position as an author, and contributions from his pen will not fail to receive respectful attention from the medical profession of this country. The present work is not, it is true, strictly an original work; that is to say, it is not made up of personal observations, nor does it profess to offer any new inductions, or novel doctrines. In the language of the author, it "aims at no other excellence, and no higher merit, than that of being a methodical and compendious summary of the actual state of our knowledge upon these most common and important diseases." [Viz. Typhoid and Typhus fevers, preface first edition, retained.] But it is by no means to be regarded in the light of a mere compilation, or a digest of the opinions of others. The actual state of our knowledge of fevers is not a matter to be determined by mere bibliographical research. It is a task, than which few in the whole range of medical inquiry are more profound and difficult, to determine the actual state of our knowledge of fevers, or, in other words, what doctrines are most consonant with the present condition of medical science. To treat upon this subject requires, in addition to the collection and arrangement of facts to be gathered from various sources, investigation, experience, ratiocination. And in bringing these requisites to bear upon the subject, originality is by no means compromised by availing oneself of the labors of others, or by arriving at conclusions similar to those at which others had already arrived. The doctrines which Prof. B. advocates have, in the main, been before propounded, but the work before us bears sufficient internal evidence that the author is an independent inquirer, thinker and observer, and, as such, it bears the impress of originality, albeit, as respects data and doctrines, it may not have the advantage of priority. While the work is reliable as presenting in a concise, methodical shape the results of the labors of Louis, Gerhard and others, it will also commend itself to the American reader not less, as containing the views of its author.

With these prefatory remarks, we proceed to the first half of the work, viz: the portion treating of Continued Fever. We have, as yet, had time to examine only this portion of the work. We shall not, however, undertake to present an analysis of the portion treating of Typhoid and Typhus fevers. The natural history of these affections could not be condensed within the limits of an analytical review, without doing injustice to the subject. For this we must refer our readers to the work itself. We purpose to confine ourselves chiefly to some of the positions advocated by Prof. B., which are at present matters of controversy, and respecting which we have the misfortune to differ from the able author.

Dr. Bartlett recognizes two species of Continued fever, which he designates *Typhoid* and *Typhus*. The subject of a third species, which is now commonly distinguished as Inflammatory, he dismisses, summarily, with the remark that he has "no knowledge of any such disease." Negative testimony, however, is entitled to little or no weight in opposition to positive testimony by equally competent witnesses as to the existence of a disease, as of any thing else; and with respect to the occurrence of a form of fever characterized by the symptoms which different writers have described as belonging to Inflammatory fever, as it seems to us, the evidence is altogether too important to be thus, we may almost say, flippantly disposed of. A fever to which a term of similar signification

(*Kausos*) was applied, was described by Hippocrates. It was described by Cullen under the denomination of *Synocha*. Dr. Christison, and other late English writers, have applied to it the adjective inflammatory, and German, French, and Italian authors have designated it by terms, which, in their languages, respectively, have a similar signification. The title is objectionable, although it has some significancy as descriptive of the distinguishing phenomena. It is objectionable, because it implies that the disease partakes of the essential nature of inflammation, while it is essentially a fever. Whether the form of fever called inflammatory differs in essence, or in its causes, proximate and remote, from the form which the author calls Typhoid, may be doubted ; but that a form of fever presented in occasional epidemics, and in sporadic cases, having the traits which are described as belonging to inflammatory fever, rests upon an amount of collective testimony which is not to be turned aside by the declaration of a few observers that they have no knowledge of it. Want of knowledge respecting it may be a sufficient reason for omitting to consider it, but, assuredly, not for implying that it has no existence.

*Part First* of Dr. Bartlett's treatise is devoted to typhoid fever, and occupies 168 pages of the work. Under the name of typhoid, Dr. B. includes all continued fevers which are not typhus. This is the latitude of signification by which this term is now very generally used. Some, however, [Bigelow & Holmes' edition of Marshall Hall on Diagnosis] apply the term to denote an intermediate grade between typhus and common continued fever. Considered in the former sense, the term is certainly an unfortunate selection. It signifies *typhus-like*, while it is contended that the disease is quite dissimilar, and, indeed, essentially *unlike* typhus. We prefer the more homely phrase, *common continued fever*. In our view, the latter is more appropriate and significant. It expresses, what is really and emphatically true, that it is the form of continued fever which is commonly presented in localities where miasmatic fevers are not predominant—typhus being comparatively unfrequent.

The symptomatology of typhoid fever is concisely, and lucidly presented, occupying about twenty-five pages of the work. The numerical researches of Louis furnish the basis of this descriptive history, as they have to most writers on the subject, since the publication of the admirable treatise on fever by that distinguished pathologist. No stronger testimony of the value of the numerical method, as furnishing symptomatological data, could be adduced, than the fact that writers who differ from Louis on important pathological questions, and are not disposed to eulogise that method of investigation, nevertheless are ready to adopt the results of the method as applied, in his hands, to the history of fever.

As it seems to us, not the least, if not the most important of the advantages of statistics to the phenomena of diseases, relates to their history and diagnosis. A sufficiently large collection of cases of any disease, faithfully recorded by properly qualified observers, accurately collated, carefully compared as respects each event or symptom of importance, and the results expressed in figures, contains the materials for a descriptive history of that disease the most complete, reliable and available that the present state of science admits of. The history, indeed, might, in this way, be rendered perfect, were it not that diseases at different times and places exhibit different phases, so that a description of any disease which is in all particulars exact as respects the cases investigated, can never continue to be rigidly applicable to the same disease in all localities, and for all time. This consideration is important to be taken into account as qualifying the perpetuity and universality of the results of the numerical analysis of cases occurring at any stated period. It is, perhaps, more applicable to fever than to most other diseases. The same consideration, however, applies with far greater force to descriptions of disease based upon the prominent events compiled from unrecorded, but merely recollected experience.

The latter are necessarily imperfect, and often incorrect. Compared with a history deduced from numerical analysis, they are like the charcoal sketches of the caricaturist contrasted with finished portraitures by the accomplished limner.

Leaving this digression, and returning to the work before us, we find nothing in the chapter devoted to the symptoms of typhoid fever which suggests critical remark. Our readers are, of course, familiar with the general course of events which appertain to the progress of the disease.

We pass by, also, the third chapter, the subject of which is the anatomical lesions. These we have considered, together with some of the pathological questions therein involved, in a former number of this Journal. [See vol. 3d, Nos. for Dec. and Jan.]

*Chapter Third* treats of the causes. On this subject the present work is more full than the first edition. The author addressed letters to medical gentlemen residing in different sections of our country, and submits the information contained in their replies, which appear to show that this form of fever prevails more extensively than has been heretofore generally supposed. It is the prevailing fever of the Eastern States, where, for the most part miasmatic fevers are unknown. But there is reason to think that in the Western, Middle, and Southern States, it has always existed, more or less, intermingled with miasmatic fevers, and very frequently confounded with the latter under the name of bilious remittents.

It is a question of interest and importance, in how far are typhoid fever and remitting fever essentially distinct. Without discussing this question (which the limits and scope of this article do not admit of) we may remark, that we entertain no doubts that the two forms of fever are presented in combination in places where miasmatic influences exist: in other words, that cases occur in which the characters of both are commingled, constituting a hybrid fever, which cannot be assigned readily to either nosological division. We have records of cases of that description, which have fallen under our own observation.

On the subject of contagion, the author does not commit himself. He adduces the opinions of different individua s which are discordant, and thinks further observations are required to settle this mooted question. On this point we would ask what stronger evidence can be desired than the following: A stranger arrives at a small settlement with the symptoms of typhoid fever, which proves fatal. Before his arrival, the persons of the settlement were healthy—no fever has prevailed there—and typhoid fever is not the kind of fever usually met with in that locality. A fortnight after the arrival of the stranger, several persons in the same house are attacked with typhoid fever. Successively, others, residing in houses located nigh at hand, become affected. The disease extends into families, the members of which are brought into contact with the sick; families which were not in close intercourse with those among whom the disease prevails, escaping. Finally more than one half of the entire population suffer an attack of the fever. This is a brief summary of the facts of an epidemic which occurred in the little settlement of North Boston, near this place, a report of which, by the editor of this Journal, was published in the American Journal of Sciences, July, 1845. The population of the settlement consisted of only forty-three souls, and, of these, twenty-eight were affected with the fever, between Oct. 19th and Dec. 7th. This is but a single example, to which we refer, because it happened to come under our notice. Other instances, more or less analagous, have been reported. Assuredly, such facts furnish all the proof of contagion that could be expected to exist. That contagion enters into the ætiology of typhoid fever seems to us clearly established: that it involves contagion as an exclusive source is another question. Nor does it follow that it is equally contagious at all times and places.

Due regard to space compels us to forego comments on the succeeding chapters in order. We pass at once to the second part of the work, the subject of which is *Typhus Fever*. This part occupies about 130 pages. The point to which the remainder of our remarks must be confined, has given rise, of late years, to much discussion—*the identity of typhoid and typhus fevers*. The author bestows upon this point considerable attention, under the head of *diagnosis* of typhus.

Let us first ask what is really involved in the inquiry whether typhoid and typhus fevers are identical. Do we mean that it is questionable whether there be a propriety in the division of continued fevers into the two forms expressed by the terms Typhus and Typhoid? Whether this be questionable or not, this is not the question which lies at the bottom of the discussion. The true point of issue is this: Is the special affection of Peyer's glands, described by Louis as the "anatomical characteristic" of typhoid, a distinctive mark of that form of fever, as distinguished from typhus? It will serve to disembarrass the subject of its apparent perplexity, to appreciate clearly the real source of discrepancy. If it be asked whether there are not grounds for distinguishing certain cases of fever as typhus, from other cases which may be called typhoid, or common continued fever, nearly all practitioners will, at once, reply in the affirmative. The title, typhus, has been used with different degrees of latitude, it is true, by different writers; but it is as old as Hippocrates, and has always been used in somewhat the same sense as now. But after Louis professed to find, as a constant lesion of the fevers of Paris, an affection of Peyer's glands, and subsequent observers in Great Britain, and in this country, found that in some cases of fever this lesion was present, and in others it was absent, the inquiry arose if the presence or absence of this lesion does not afford a fundamental point of distinction between two forms of fever. Dr. Bartlett and others have taken the position that this lesion is invariably present in one form (typhoid,) and that it is invariably absent in typhus. Hence, as we have said, the question now is, not whether there is an intrinsic propriety in calling certain fevers typhus, and others typhoid, common continued, or by some other name; but the question really is, whether an affection of Peyer's glands is a reliable test of the latter, as distinguished from the former. In a previous number of this Journal, [Vol. 3, No. for Jan.] we have endeavored to show from Louis' own collection of cases, that the lesion of Peyer's glands which he regards as constant and characteristic, is not present in all cases of typhoid fever, and that it is not peculiar to typhoid fever. Observations since the publication of Louis' work have abundantly established that all cases of fever exhibiting during life the phenomena of typhoid fever, do not present, on dissection, this lesion, and, on the other hand, cases exhibiting during life all the characters of typhus fever, may present, in dissection, this lesion. With regard to the latter, (upon which the mooted question turns.) the report of M. H. Landouzy of an epidemic typhus fever which prevailed at Rheims, between Oct. 1839, and April, 1840, furnishes unequivocal evidence. The reader will find a summary of this report in Dr. Bartlett's treatise, in the chapter on the diagnosis of typhus. Here are over one hundred and thirty-eight cases which, beyond all cavil, were cases of typhus, judged by external, living phenomena ; while in all the autopsies made, six in number, the intestinal lesions regarded by Louis and others as characteristic of typhoid, were found. Dr. B. admits, in the present edition, that the cases of M. Landouzy throw some doubt on the question whether "typhoid and typhus fevers are clearly and unequivocally, fundamentally distinct diseases ;" an admission which is inconsistent with other passages of the work, asserting a fundamental, unequivocal distinction. But we are sorry to see an indication of want of candor in the author's comments on M. Landouzy's report, which should not escape critical notice. Speaking of that epidemic, he uses the following expression, and emphasises it by putting it in italics : "*If entire reliance is to be placed upon the observation of its historian.*" He virtually implies in this expression that there is room for suspicion as to the veracity of M. Landoury. Does he *know* any grounds for suspicion ? If so, he was bound to have stated them fairly and fully. If he has no better reason for the suspicion than that the results compromise the correctness of his own opinions, then the insinuation is ungenerous ; nay, it deserves a harsher epithet, which we leave the reader to supply. We regret to observe in other places a disposition to discredit the accuracy of the diagnosis in reports of cases at variance with the position which the author labors to sustain, while there is apparent an equal amount of laxity when the results are favorable to this position. We are far from intending to impute any want of good faith, but it is well known how often and how much the mind becomes warped by the desire to maintain opinions to which it is once fully committed.

We do not of course, profess, in this connection, to discuss at length the question as to the specific distinctions which obtain between typhus and typhoid fevers, in the sense in which they are held by Dr. Bartlett and others. We have only offered some desultory remarks, with the view of placing the question in its right aspect, and furnishing suggestions for the reflections of our readers. We will merely add a few propositions embodying conclusions to which we have been led by our own reflections on the subject :—

1. The internal lesions described by Louis as the " anatomical characteristic" of typhoid fever do not constitute an exclusive trait of a species of fever distinct from typhus. That these lesions are very generally present in cases of fever presenting the train of symptoms described as belonging to typhoid fever, may be admitted. It may also be admitted that they are more rarely present in cases presenting the symptoms characterizing typhus. But they are sometimes present in well marked cases of typhus, i. e. cases in which all the external phenomena of typhus are exhibited.

2. Agreeably to the facts contained in the foregoing proposition, there can be no propriety in instituting these lesions as a sure test of a species of fever distinct from typhus. To do this, and consequently consider all cases in which these lesions are revealed by dissection, to have been cases of typhoid fever, whatever may have been the symptoms during life, must necessarily lead to confusion and error. It involves an obvious *petitio principii*. If these lesions are regarded as constituting an unfailing *post-mortem diagnostic* of typhoid fever, it follows that all fatal cases which present the lesions are cases of typhoid, as a matter of course; but this is obviously begging the question. It seems to us Louis and his followers have fallen into this logical error, and that it may be proved from Louis' own observations that the " anatomical characteristic" was a premature generalization, which subsequent observations have still more conclusively disproved.

3. The divisions of continued fever in the present state of knowledge, are to be based chiefly on differences in external phenomena, in other words the events incident to the febrile career. Analysis and comparison of cases of continued fever, occurring at different periods, and places, will furnish sufficient data for divisions corresponding, for the most part, with those which are generally recognized by writers and practitioners, viz: Inflammatory, Common continued or typhoid, and Typhus. The point which we would indication in this proposition is, that the divisions are to be based for the present, on facts appertaining to the living body, not on lesions found after death!

4. It seems, of late, to be overlooked, that in the study of different forms of continued fever, we are to take into account their points of resemblance as well as differences, in arriving at an opinion as to the relations which they sustain to each other. This consideration is important with reference to the question whether the different forms are but *varieties* of a single disease, or distinct species of disease—in other words, distinct diseases.

If we place the symptoms of the so-called typhoid and typhus fevers together, being careful to note what particulars appertaining to the febrile state are common to both, as well as what are in contrast, in our opinion we must arrive at the conclusion, that, essentially, they are identical: that is to say, that continued fever is one disease, presented, at different times and places, under modifications which are sufficiently uniform to admit of classification into nosological varieties, but which do not denote distinctions in essence.

We give the foregoing views in an aphoristical form merely for the sake of brevity. The reader will, of course, take them for what they are worth. Each proposition admits of a wide range of discussion, for which we have not at present time, nor space, even were we willing to incur the risk of exhausting the patience of the reader.

In conclusion, we transfer to our columns the following tabular summary, exhibiting the leading points of distinction between typhus and typhoid fever, side by side:—

| Typhoid Fever. | Typhus Fever. |
|---|---|
| 1. *Mode of Access.* — More generally gradual, insidious, and creeping, than in typhus, | 1. More frequently sudden and formal than in typhoid fever. |
| 2. *Heat of Skin.* | 2. More frequently burning and pungent during the early stages, than in typhoid fever. Fuliginous flush of face more common than in typhoid fever. |
| 3. *Mind.* — Delirium and other cerebral symptoms, coming on, and increasing gradually, after the first week, more generally than in tyhus. | 3. Cerebral symptoms, especially dullness and stupor, more strongly marked at the outset of the disease than in typhoid fever. |
| 4. *Bowels.* — Diarrhœa, with thin liquid discharges, very common. Gurgling on pressure over region of cæcum Meteoric distention or rigidity of abdomen. Griping pains common. | 4. Spontaneous diarrhœa rare. Discharges from the bowels not liquid. No gurgling on pressure over region of cœcum. Meteoric distension very rare. Griping pains rare. |
| 5. *Emaciation.* — More common and greater than in typhus. | 5. |

| Typhoid Fever. | Typhus Fever. |
| --- | --- |
| 6. *Epistaxis.* — More common than in typhus. | 6. |
| 7. *Hemorrage from the Bowels.* — Quite common. | 7. Very rare, Does it ever occur ? |
| 8. *Cutaneous Eruptions.*—Bright, scanty, rose-colored eruption , slightly elevated above the surrounding skin : readily disappearing on pressure ; mostly confined to skin of chest and abdomen. | 8. In many cases, especially grave ones, more abundant petechial eruption ; not disappearing on pressure:—in other cases, no eruption. |
| 9. *Eschars.* — More common than in typhus. | 9. |
| 10. *Lesions.* — Peyer's glands always altered ; generally ulcerated. Mesenteric glands reddened, enlarged and softened. Spleen more frequently enlarged and softened than in typhus. Ulceration of the pharynx and œsophagus more common than in typhus. Large intestines more frequently distended with gas than in typhus. | 10. Peyer's glands, and mesenteric glands healthy. Blood more generally dark and grumous. Dark engorgement of vessels and sinuses of brain more constant than in typhoid fever. |
| 11. *Causes.* — Confined to no geographical localities. Prevailing constantly and extensively amongst scattered, cleanly, well fed, and well sheltered rural populations. Occasionally and moderately contagious. More frequently sporadic than typhus. More generally limited to the early and middle period of life than typhus. | 11. Limited to certain geographical localities Generally confined to crowded, filthy, and poorly ventilated habitations. Under such circumstances eminently contagious. Occurring much more frequently after the thirty-fifth year of life than typhoid fever. |
| 12. *Duration.*—Average duration somewhat greater than typhus. Prolonged to the fortieth or fiftieth day much more frequently. | 12. Terminating fatallv, or in recovery within the first ten days much more frequently than typhoid fever. |
| 13. *Effect of Remedies.*—Bearing depletion better than typhus. | 13. Requiring more active stimulation than typhoid fever. |

With reference to the foregoing summary, it is to be remarked, that the facts relating to typhoid fever are chiefly those deduced from the numerical observations of Louis. We have already expressed our sense of the high claims which these observations possess. They are doubtless the best our science possesses: but it is to be borne in mind that they are not perfect, inasmuch as the disease is subject to variations. Excellent and complete as they are, it does not follow but that had the number of cases been more numerous; had they occurred at London, or Edinburgh, or New York, instead of at Paris; had the patients been subjected to different methods of treatment, instead of the same method, and had they been private instead of public cases, the results of the analysis would have not been precisely what they are. It is to be remarked, also, that the symptoms of typhus, in a great measure, are derived from the observations of Gerhard, which are less to be considered as constituting a standard group of symptoms, because they are deduced from the study of a single epidemic.

If the reader, however, will weigh well the points of contrast as presented in the table, rejecting, as he must do, the points of distinction which relate to Peyer's glands, and summing together all the points of resemblance as well as the points of distinction, he will find, as we think, abundant reasons for concurrence in the correctness of the last of the propositions which we have submitted.

We presume it is quite needless to assure the reader that we do not profess in this article to have reviewed Dr. Bartlett's treatise. We were the less called upon to do this, as the portion to which our remarks have been confined, is but a second edition of what has been for some years before the profession. We have availed ourselves of the occasion to indulge in a brief sketch of some of the views which we have been led to form on the subject of Continued fever, the chief design being that they may serve as suggestions for the reflections of those who may honor our desultory remarks by a perusal. We will only add, that we regard the work by Dr. Bartlett as a creditable and valuable contribution to our stock of native productions in medical literature, and we hope it will have an extensive circulation.

